# Supplementary material for: Buprenorphine Versus Methadone in Female New Zealand White Rabbits Undergoing Balanced Anaesthesia for Calvaria Surgery
Source: Animals (Basel). 2025 Jun 22;15(13):1843. doi: 10.3390/ani15131843 (PMC12249090; doi:10.3390/ani15131843)
Supplement: Supplementary file 1 [file animals-15-01843-s001.zip › Supplementary Material_Sedation Score (modified by Raekallio et al., 2002).pdf]

Rabbit ID ……………………… Inclusion number in the study ……………………………… Ethical approval: 29384

1st evaluation 15' 2nd evaluation 20' 3rd evaluation (after 2nd injection)

Sedation score

1) Posture

0 = no change

1= lying sternally, head down, reactive to touch

2= lying sternally, head down, can be rolled in lateral recumbency

3= lying laterally

2) Resistance to being rolled in dorsal recumbency:

0 = impossible

1= strong resistance

2 = mild resistance

3 = no resistance

3) Muscle tone

0 = normal

1 = mild muscle relaxation (limb mild relaxed)

2 = moderate muscle relaxation (reduced nuchal tone, limbs relaxed)

3 = total muscle relaxation

4) Palpebral reflex

0= normal

1= decreased

2= absent

Decision tree:

With sedation ≤ 7 (1-4), wait a further 5 minutes and reevaluate (2nd evaluation).
With no changes, inject 5 mg kg^{-1} ketamine and 0.02 mg kg^{-1} dexmedetomidine IM.
Repeat the evaluation after 10 minutes.
